# IL-6 and Surgical Outcomes in Carotid Endarterectomy: A Systematic Review

**DOI:** 10.3390/medsci13040325

**Published:** 2025-12-18

**Authors:** Antónia Rocha-Melo-Sousa, Márcio Brazuna, Carmen Tavares, Sai Guduru, Mariana Fragão-Marques, João Rocha-Neves

**Affiliations:** 1 Faculty of Medicine, University of Porto, 4200-319 Porto, Portugal; up202306224@edu.med.up.pt; 2 Faculty of Medicine and Biomedical Science, University of Algarve, 8005-139 Faro, Portugal; a55054@ualg.pt; 3 Nova Medical School, 1169-056 Lisboa, Portugal; carmenmanuelt@gmail.com; 4 Department of Internal Medicine, Mosaic Life Care, University of Missouri–Kansas City, St. Joseph, MO 64506, USA; saisivaramguduru@gmail.com; 5 Department of Immuno-Physiology and Pharmacology, ICBAS School of Medicine and Biomedical Sciences, University of Porto, 4150-180 Porto, Portugal; marianaif.rm@gmail.com; 6 Department of Biomedicine, Unit of Anatomy, Faculty of Medicine of University of Porto, 4200-319 Porto, Portugal; 7 RISE-Health, Departamento de Biomedicina, Faculdade de Medicina, Universidade do Porto, Alameda Prof. Hernâni Monteiro, 4200-319 Porto, Portugal; 8 Department of Vascular Surgery, Unidade Local de Saúde–Alto Ave, 4835-044 Guimarães, Portugal

**Keywords:** carotid artery diseases, atherosclerosis, cytokines, biomarkers, prognosis, risk factors

## Abstract

**Background:** Interleukin-6 (IL-6) is a key inflammatory cytokine implicated in atherosclerotic plaque progression and carotid vulnerability. Although elevated IL-6 levels have been linked to cerebrovascular risk, its prognostic value in patients undergoing carotid endarterectomy (CEA) remains undefined. This systematic review aimed to investigate the available evidence on the relationship between IL-6 levels, surgical outcomes and mechanistic evidence in CEA patients. **Materials and Methods:** The review followed the PRISMA statement and AMSTAR-2 critical appraisal guidelines, with the protocol registered on PROSPERO (CRD420251120023). PubMed/MEDLINE, Scopus, and Web of Science were systematically searched up to July 2025 using the terms “interleukin-6” and “carotid endarterectomy”. Original studies in humans assessing IL-6 in relation to clinical outcomes after CEA or mechanistic evidence were included without language or date restrictions. Study quality was evaluated using the Cochrane Risk of Bias 2 and NHLBI tools, and evidence certainty was appraised using the GRADE framework. Given the heterogeneity of studies, only a qualitative synthesis was performed. **Results**: From 1232 records identified, 13 studies encompassing 1396 patients met the inclusion criteria. Most were prospective observational cohorts, with a mean participant age of 68.52 years and 81.16% male predominance. Perioperative stroke and mortality rates were uniformly low (≤2%), consistent with contemporary registry data. Across studies, elevated IL-6 levels—whether systemic or plaque-derived—were consistently associated with symptomatic carotid disease, plaque vulnerability, and adverse long-term outcomes. However, not all studies presented quantitative data on IL-6 levels, limiting the ability to draw definitive prognostic conclusions. **Conclusions**: Current evidence supports a mechanistic link between IL-6–mediated inflammation and carotid plaque instability, yet robust clinical validation in surgical populations is lacking. Future large-scale, prospective studies incorporating IL-6 measurement are warranted to establish its prognostic utility, guide anti-inflammatory therapeutic strategies, and refine postoperative risk stratification in patients undergoing CEA.

## 1. Introduction

Carotid endarterectomy (CEA) remains the gold standard intervention for patients with symptomatic or high-grade asymptomatic carotid stenosis, effectively reducing the risk of recurrent stroke [1]. Despite advances in surgical technique and perioperative care, early postoperative complications such as stroke, myocardial infarction (MI), and death remain important concerns, especially in older and comorbid patients [2]. In contemporary trials and registries, 30-day perioperative stroke and death rates range between 1–3% [3], but the long-term risk of adverse vascular events remains significant, particularly in patients with residual systemic inflammatory activity. Recurrent stroke, restenosis, and cardiovascular mortality continue to occur in the years following CEA, suggesting that mechanical plaque removal alone may not address the underlying biological drivers of disease [4]. Importantly, elevated levels of inflammatory markers, such as C-reactive protein (CRP) and IL-6, have been linked to worse outcomes after vascular interventions [5], although few surgical studies have specifically evaluated their prognostic value in the setting of CEA.

Carotid atherosclerosis is a progressive inflammatory disease characterized by endothelial dysfunction, lipid accumulation, immune cell infiltration, and fibrous cap formation within the carotid arteries, ultimately leading to plaque development and luminal narrowing [6]. This process is orchestrated by a complex interplay of pro-inflammatory cytokines and chemokines, among which interleukin-6 (IL-6) plays a central role. IL-6, a pleiotropic cytokine produced by endothelial cells, macrophages, and smooth muscle cells, contributes to all phases of atherosclerotic plaque development—from endothelial activation to thrombogenic remodeling [7]. Elevated systemic IL-6 levels have been independently associated with carotid intima-media thickness progression and increased plaque vulnerability in large-scale prospective cohorts, indicating its potential utility as a biomarker for subclinical vascular inflammation and cerebrovascular risk [8]. Furthermore, IL-6 signaling through the JAK/STAT3 pathway promotes local inflammation, smooth muscle cell proliferation, and matrix degradation, key contributors to plaque rupture and thromboembolic complications [9].

This systematic review aimed to evaluate the relationship between IL-6 and clinical outcomes and mechanistic evidence following CEA. The primary objective was to assess the association between IL-6 and the incidence of stroke in the postoperative period. Despite this, as highlighted by the heterogeneity and modest sample sizes across the included cohorts, this objective must be interpreted as exploratory rather than confirmatory. The available evidence does not allow for definitive causal inference, and our findings instead underscore the need for adequately powered, prospective studies specifically designed to evaluate IL-6 as a prognostic marker in the immediate postoperative period. Secondary objectives included examining the relationship between IL-6 and major adverse cardiovascular events, other cerebrovascular and cardiovascular complications occurring in the long-term (>30 days) follow-up after CEA, as well as mechanistic evidence. We acknowledge that a 30-day threshold only represents the conventional distinction between perioperative and post-discharge outcomes and does not constitute true long-term surveillance. Because most included studies reported follow-up of limited duration, we adopted the reporting structure used by the original cohorts. Nevertheless, we emphasize that multi-year follow-up is essential to fully understand the prognostic relevance of IL-6, particularly in restenosis and cardiovascular recurrence.

## 2. Materials and Methods

This systematic review was conducted in accordance with the Preferred Reporting Items for a Systematic Review and Meta-analysis (PRISMA) Statement and the AMSTAR-2 critical appraisal tool [10,11]. An institutional review board’s ethical approval was not obtained due to the nature of this study. The review protocol has been registered at Prospero (reference: CRD420251120023).

### 2.1. Selection Criteria

Inclusion criteria consisted of all original articles performed in humans (except for systematic reviews and case series under 20 patients) that evaluated interleukin-6 (IL-6) levels in patients undergoing carotid endarterectomy (CEA) and their association with clinical outcomes or mechanistic evidence. No exclusion criteria based on the publication language or date were applied. Other exclusion criteria included pediatric populations, patients without confirmed carotid disease, patients undergoing synchronous cardiac surgery, carotid stenting, or carotid reintervention. Case reports, editorials, letters, conference abstracts (unless with sufficient data) were also excluded.

### 2.2. Search Strategy

A systematic search was performed in three databases—PubMed/MEDLINE, Scopus, and Web of Science, in July 2025. The search strategy combined the terms (“interleukin-6” OR “IL-6” OR “interleukin 6”) AND (“carotid endarterectomy” OR “CEA”), as detailed in Supplemental Table S1. References from included studies and relevant systematic reviews were manually screened for additional eligible articles.

### 2.3. Study Selection and Data Extraction

After duplicate removal, two authors (ARM and MB) independently participated in study selection; any disagreement was solved by the intervention of a third author (JRN). First, studies were selected by title and abstract, and the remaining ones were eligible for full-text assessment. Efforts were made to contact the authors to obtain the full texts that were not publicly available. The selected studies were carefully revised to avoid repeated populations.

Data from included studies were independently extracted by two authors (ARM and MB) using a purpose-built form, which included the year of publication, country, center of recruitment, study design, recruitment time, number of participants undergoing carotid endarterectomy, participants’ age and gender distribution, frequency of cardiovascular comorbidities, and carotid symptomatic status.

### 2.4. Assessment of Study Quality

Concerning qualitative assessment, the Cochrane Risk of Bias-2 tool was used for randomized clinical trials [11] and the National Heart, Lung, and Blood Institute (NHLBI) Study Quality Assessment Tool for observational cohort and cross-sectional studies (2021) [12]. This assessment was independently performed by two authors (ARM and MB), and when disagreements were observed, decisions were made by consensus after a third-party review (JRN). The quality of evidence for the included articles was evaluated using the Grading of Recommendations, Assessment, Development, and Evaluation (GRADE) approach. Articles were classified into four levels of quality (high, moderate, low, and very low) [13].

### 2.5. Quantitative Synthesis

No quantitative meta-analysis was performed due to the heterogeneity of eligible studies, which precludes reliable pooled estimates. Instead, a qualitative synthesis was conducted, summarizing IL-6 levels in relation to surgical outcomes following CEA, including neurologic events and restenosis, as well as mechanistic evidence.

## 3. Results

### 3.1. Search Results

**Figure 1:**
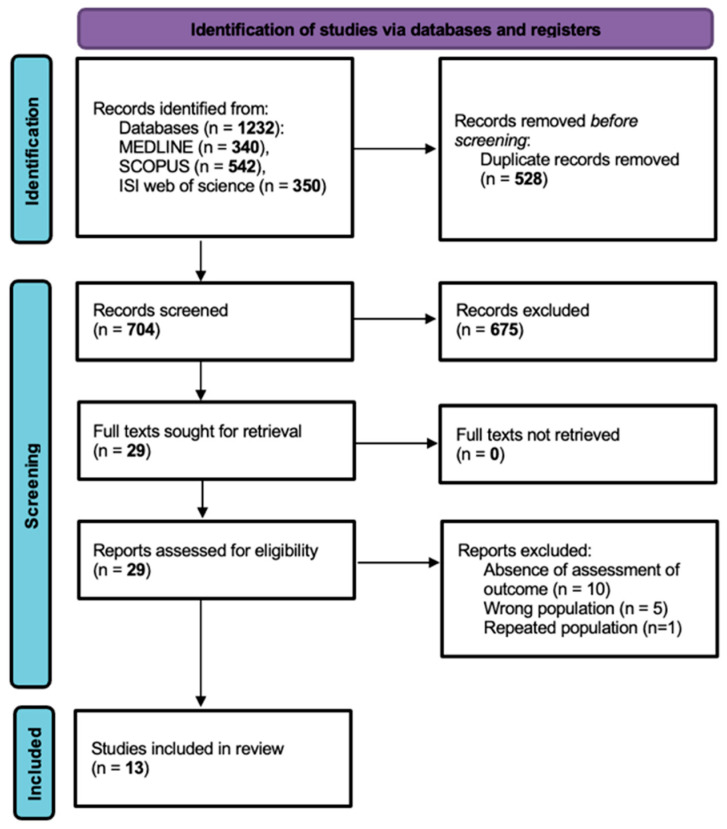
PRISMA flowchart illustrating study selection.

Firstly, 1232 records were identified from the databases (MEDLINE *n* = 340, SCOPUS *n* = 542, ISI Web of Science *n* = 350) and 528 duplicate records were removed, with a total of 704 studies selected for screening. Upon title and abstract screening, 675 records were excluded. The full texts for 29 reports were sought for retrieval and assessed for eligibility, with 16 reports ultimately excluded. The comprehensive criteria for exclusion upon full-text assessment were no outcome assessment (*n* = 10), wrong population (*n* = 5) and repeated population (*n* = 1). Thus, a total of 13 published articles were included in this systematic review (Figure 1). This systematic review was conducted and reported in accordance with the PRISMA 2020 statement.

### 3.2. Description of Studies

Of the 13 studies included in this systematic review, 10 were observational cohorts [14,15,16,17,18,19,20,21,22,23], with 3 of these being retrospective [24,25,26]. The other 3 studies included 1 randomized controlled study [15] and 1 prospective cross-sectional study [18]. The included publications were performed in 6 different countries across 3 continents. In terms of continents, ten studies were from Europe [14,15,17,18,19,20,21,22,23,24,27], two were from Asia (China) [25,26], and one was from North America (USA) [16]. A total of 1396 patients were assessed, with a minimum of 20 patients [21] and a maximum of 292 patients [19] per study. The mean participants’ age, calculated from the available data across 10 studies, was 68.52 years old. The overall percentage of male participants was approximately 81.16% (*n* = 1133) based on the total pooled sample size of 1396. Demographics and comorbidities of the populations included in the studies were gathered and are available in Table S3.

### 3.3. Main Findings and Meta-Analysis

#### 3.3.1. CEA Clinical Outcomes at 30 Days

The reported 30-day incidence of complications after carotid endarterectomy (CEA) in the included studies did not vary widely. Stroke rates ranged from 0.0% to 1.7% and were reported in 4 studies [15,16,22,27]. These same 4 studies reported combined stroke or death, which ranged from 0.0% to 2.6%, and 30-day mortality ranged from 0.0% to 1.3%. Myocardial infarction (MI) incidence was reported in only one study [27] at 1.7%, and major adverse cardiac events (MACE) were reported in two studies [22,27], ranging from 1.7% to 3.9%.

#### 3.3.2. CEA Long-Term Clinical Outcomes

Long-term clinical outcomes following carotid endarterectomy (CEA) in the selected studies demonstrated notable variability but generally reflected favorable results with low incidence of major adverse events over extended follow-up. For instance, Persson et al. [19] reported a comprehensive five-year follow-up of 160 patients, showing 52 cases (17.8%) of all-cause death, with 28 attributable to cardiovascular causes and 25 to other causes, and 9.6% incidence of stroke within the cohort Boutouris et al. [17] described a 4.1% incidence of myocardial infarction and 5.4% incidence of stroke over two years, with a subset requiring revascularization due to carotid restenosis Unic-Stojanovic et al. [22] provided some long-term data indicating isolated cases of post-operative cognitive dysfunction during extended surveillance. Across the included studies, recurrent major complications—such as mortality, myocardial infarction (MI), and cerebrovascular events—were comparatively rare, underscoring the sustained benefits of CEA for secondary prevention in appropriately selected patients.

#### 3.3.3. IL-6 and Post-Operative Stroke

Analysis of the included studies indicates that elevated post-operative IL-6 levels and increases (delta) from pre- to post-surgery are associated with a higher incidence of cerebrovascular events within the first 30 days after carotid endarterectomy (CEA). Notably, Unic-Stojanovic et al. [22] reported a delta IL-6 of 14.84 pg/mL in a cohort with one postoperative stroke and two transient ischemic attacks (TIAs), underscoring the potential prognostic significance of acute IL-6 surges for short-term neurological complications. These observations, however, derive from very small patient subsets, and the rarity of perioperative stroke inherently limits statistical power. Therefore, while the signals are biologically plausible and consistent with mechanistic data, they should be interpreted cautiously and viewed as hypothesis-generating rather than definitive. Koutouzis et al. [18] also documented two cases of early cerebrovascular events, one occurring in a patient with low baseline IL-6 and one in a patient with high IL-6, suggesting that not just absolute values but perioperative dynamics may influence risk, in line with mechanistic models of inflammation-driven plaque instability and embolic potential. Importantly, none of the included studies directly correlated plaque morphology such as lipid-rich necrotic core, fibrous cap thickness, or ulceration with postoperative stroke events. Although the wider literature robustly links these morphological features to cerebrovascular risk [28], the cohorts selected for this review did not report postoperative stroke in relation to plaque characteristics, preventing any conclusions on this association within our dataset.

#### 3.3.4. IL-6 and Major Adverse Cardiovascular and Cerebrovascular Events in Long-Term Follow-Up

While long-term (≥30 days) pooled data were sparser, select studies provided evidence of a relationship between IL-6 and adverse cardiovascular outcomes after CEA. For example, cohorts with higher post-operative or persistently elevated IL-6 experienced greater rates of carotid restenosis (Unic-Stojanovic et al. [22]: two restenosis), recurrent ischemic events, and cardiovascular mortality. Across studies, however, the definition of restenosis was inconsistent. While some cohorts defined recurrence as ≥50% luminal narrowing by duplex ultrasound, others adopted a ≥70% threshold or relied on peak systolic velocity criteria [29]. This variability makes pooled interpretation difficult. Stein et al. [16], in contrast, observed no long-term MI, stroke, or death in their group with lower delta IL-6 values (10.92 pg/mL), reinforcing an association between lower inflammation and improved long-term follow-up outcomes. These findings parallel broader atherosclerosis research, where sustained elevation of inflammatory biomarkers such as IL-6 is well established as a risk factor for cardiovascular recurrence and diminished procedural durability in large-scale analyses [30].

### 3.4. Meta-Analysis

A meta-analysis was not conducted since the included studies presented heterogeneity in the reported endpoints, hindering the possibility of adequate pooled estimates.

### 3.5. Quality Assessment

**Figure 2:**
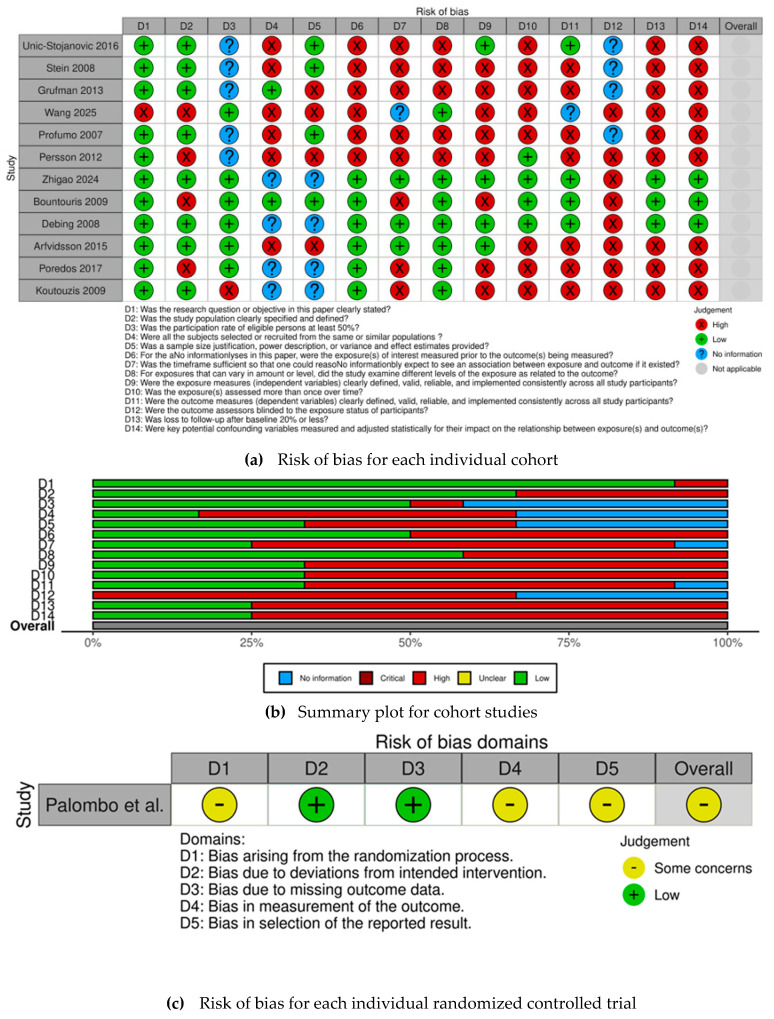
(**a**) Risk of bias for each individual cohort [14,16,17,18,19,20,21,22,23,24,25,26] (**b**) Summary plot for cohort studies. (**c**) Risk of bias for each individual randomized controlled trial [15].

The risk of bias for each selected observational cohort is individually displayed in Figure 2a, while the risk of bias for the selected RCT is displayed in Figure 2c. The overall judgement per evaluated item regarding observational cohorts is shown in Figure 2b.

All observational cohorts had an overall high risk of bias, except for Zhigao et al. [26], Bountouris et al. [17], Debing et al. [24], and Arfvidsson et al. [21]. The most frequently identified methodological shortcomings, which contributed to the high risk of bias among observational cohorts, were concentrated in several key domains—confounding, inadequate measurement, and statistical adjustment for key potential confounding variables regarding exposure assessment. The items most frequently associated with high risk of bias also included the lack of sample size justification, insufficient description of statistical power, and failure to report clear variance and effect estimates, leading to concerns about the precision of the obtained results. Conversely, there were four studies [17,21,24,26] that demonstrated a robust methodology, achieving an overall low risk of bias.

### 3.6. Publication Bias

Publication bias was not formally assessed since not enough consistent clinical endpoints were evaluated for the association with IL-6 levels in the selected studies.

## 4. Discussion

This systematic review identified 13 studies assessing outcomes in patients undergoing CEA, predominantly prospective observational cohorts. It is important to emphasize that perioperative outcomes following carotid endarterectomy are inherently multifactorial and are influenced by surgeon expertise, anesthetic technique, perioperative management, and institutional protocols. These determinants exert a substantially greater impact on early postoperative complications than any single inflammatory biomarker. Consequently, while IL-6 is mechanistically linked to vascular inflammation and plaque instability, it cannot be isolated as an independent predictor of perioperative stroke, myocardial infarction, or mortality within the current evidence base. The perioperative outcomes demonstrated a low overall incidence, not reporting the relation with IL-6 levels, hence it likely reflects advances in surgical technique and perioperative management, rather than biological modulation of inflammation. While IL-6 has been implicated in vascular injury and plaque instability, its influence is expected to affect long-term, rather than immediate, postoperative outcomes. Long-term follow-up data revealed stroke and mortality incidences approaching 17.8%, directly related to high IL-6 levels, suggesting that biological risk persists beyond successful revascularization [19].

Additionally, IL-6 levels are highly variable and influenced by several patient-related, environmental, and procedural factors, including age, comorbid inflammatory conditions, baseline metabolic status, medication use, and the acute inflammatory response triggered by surgical trauma itself. This broad biological variability makes it challenging to attribute postoperative clinical outcomes specifically to IL-6, particularly in small cohorts with limited event numbers.

Unic-Stojanovic et al. [22] reported a substantial IL-6 increase (delta ~14.8 pg/mL) concurrent with stroke and transient ischemic attack events, underscoring IL-6 as a dynamic predictor of acute cerebrovascular risk. In line with the findings of Kragsterman et al. [31], carotid endarterectomy induces a localized cerebral inflammatory and coagulation-fibrinolysis response consistent with ischemia–reperfusion injury, although this process appears to occur independently of CD40–CD40L pathway activation [31]. Beyond early events, select studies linked persistently elevated IL-6 to increased major adverse cardiovascular events (MACE), myocardial infarction, restenosis, and mortality over longer-term follow-up, suggesting that it persists as a risk factor beyond successful revascularization [17,19].

While a direct causal relationship between IL-6 and adverse post-CEA outcomes has not yet been established, accumulating mechanistic and longitudinal evidence indicates that IL-6 plays a pathophysiologically central role in vascular inflammation, endothelial dysfunction, and plaque instability [30]. Elevated systemic and plaque IL-6 levels have been consistently associated with carotid plaque severity and vulnerability [32,33]. These findings suggest that IL-6 is most likely a central player in the inflammatory cascade contributing to ongoing vascular injury and adverse events after CEA. Similarly, a prospective longitudinal cohort study evaluating 210 Japanese patients with vascular risk factors but without previous major cardiovascular events at baseline from Okazaki et al. [34] demonstrated that higher IL-6 concentrations independently predict progression of carotid intima-media thickness over 9 years, reinforcing its role as a driver of ongoing vascular injury. At the tissue level, IL-6 and its receptors are highly expressed within carotid atherosclerotic plaques, particularly in symptomatic lesions, linking local inflammation with systemic cytokine activity [33,35].

Mechanistically, IL-6 promotes endothelial dysfunction, smooth-muscle proliferation, and thrombogenic remodeling through JAK/STAT3 signaling [36], all of which contribute to plaque instability and restenosis after CEA. Therefore, the long-term stroke and mortality rates observed in the included cohorts may partly reflect persistent IL-6–driven inflammatory activity, emphasizing that the benefit of CEA depends not only on mechanical plaque removal but also on the modulation of the patients’ systemic inflammatory state [17,18,37].

Finally, although several large epidemiologic studies such as the Cardiovascular Health Study [30] and Tromsø Study [32] have demonstrated that circulating IL-6 predicts carotid atherosclerosis progression and plaque vulnerability, few surgical series have translated these findings into perioperative prognostic applications. The review also revealed substantial heterogeneity in outcome reporting and follow-up duration. Heterogeneity across studies further limits interpretability. The timing of IL-6 sampling, assay methodology, definition of clinical endpoints, and degree of statistical adjustment varied considerably across cohorts. Such variability precluded quantitative synthesis and prevented any reliable estimation of effect size. This heterogeneity reflects the lack of standardized IL-6 measurement protocols in vascular surgical research and represents a key barrier to establishing its prognostic utility. While short-term (30-day) stroke and mortality rates were uniformly low (typically <2%), in line with registry data from the CREST and NASCET eras [4,38], the long-term outcomes were infrequently and inconsistently reported. Only two studies provided extended follow-up beyond two years: one demonstrated a 17.8% all-cause mortality over 5.2 years [19], while another reported 6.8% mortality at 2 years [17]. This scarcity of prolonged follow-up is a major limitation, as IL-6–mediated inflammatory pathways are more likely to exert prognostic influence over years rather than months. The absence of multi-year IL-6 surveillance prevents a robust evaluation of its long-term predictive value in restenosis, recurrent stroke, and cardiovascular mortality events. Additionally, most studies did not employ Kaplan–Meier survival reporting, limiting the ability to assess long-term outcome trajectories.

Despite a comprehensive search strategy yielding 13 eligible studies, a critical finding was the heterogeneity in the reported endpoints, hindering the possibility of adequate pooled estimates. While several studies collected inflammatory markers such as CRP or leukocyte counts, they did not present quantitative data on IL-6 levels. This absence likely reflects both historical and methodological factors. First, most clinical outcome studies in CEA have traditionally focused on surgical safety and technical efficacy rather than molecular inflammatory profiling, since perioperative stroke and death rates are difficult to correlate with single biomarkers in small cohorts. [39] Second, standardization of IL-6 assays and timing of sampling (systemic versus plaque-level) remain inconsistent across vascular studies, limiting comparability and reproducibility [40,41].

In fact, recent evidence positions IL-6 as a promising early biomarker for predicting post-operative outcomes, showing distinct advantages compared to traditional inflammatory markers such as CRP. IL-6 rises rapidly following surgery, with peak concentrations typically observed within the first 24 h [42], enabling earlier identification of patients at risk for severe post-operative complications, such as infection, organ dysfunction, or delirium, when compared to the slower kinetics of CRP, which generally peaks at 48–72 h post-operatively [43]. According to Huang et al. [44], IL-6 is upstream to CRP in the inflammatory cascade, hence proving to be a stronger early predictor of peri-operative outcomes. Studies consistently demonstrate that higher IL-6 levels on the first post-operative day are significantly associated with subsequent morbidity, longer hospital and intensive care stays, and worse disease-free survival, while CRP, although still valuable, serves as a later-stage indicator [45,46].

This systematic review is subject to several limitations inherent in the available literature. First, most were observational cohorts, which, as assessed, carried an overall high risk of bias. Furthermore, the study populations often lacked explicit sample size justifications or power descriptions, contributing to low precision and potential underestimation of rare but clinically relevant outcomes such as perioperative stroke or death. This limitation, however, is a direct reflection of the current state of evidence, and our rigorous PRISMA-guided methodology ensured that all existing evidence was included, thereby minimizing risk of under or overestimating the evidence base.

Most importantly, the included studies were not designed a priori to evaluate IL-6 as a prognostic biomarker, and the available datasets were underpowered to detect associations with rare perioperative outcomes such as stroke and death. The limited sample sizes and consistently low event rates mean that any observed associations or absences must be interpreted with caution. Thus, the available evidence should be viewed as hypothesis-generating rather than clinically actionable. Given these methodological limitations, proposing IL-6 as a clinically actionable biomarker in the perioperative setting is premature. While mechanistic and epidemiologic data strongly support its role in atherosclerotic inflammation, the current clinical literature lacks the standardization, sample size, and longitudinal biomarker design necessary to establish IL-6 as a risk-stratification tool in CEA. Future multicentered prospective studies with predefined biomarker endpoints are required to bridge this gap.

Substantial heterogeneity was observed across cohorts in terms of patient demographics, comorbidity burden, perioperative management, and surgical technique—the latter often varying by institution and not explicitly detailed. Moreover, inconsistency in defining and reporting short- and long-term outcomes, including stroke, mortality, and restenosis, prevented a valid quantitative meta-analysis. Similar methodological variability has previously been highlighted in large-scale vascular reviews and registries [47]. Despite strong mechanistic evidence linking IL-6 to plaque instability, vascular inflammation, and long-term atherosclerotic progression [30,32,40], clinical studies have yet to incorporate IL-6 as a prespecified predictor in surgical cohorts.

Nonetheless, this review has significant strengths. It presents a comprehensive synthesis of the most contemporary literature on outcomes and mechanistic evidence in CEA patients and IL-6. Furthermore, it was conducted according to a pre-registered, PRISMA-compliant protocol with transparent risk-of-bias assessment across all included studies, ensuring reproducibility and methodological rigor. Ultimately, this work is instrumental in exposing the need for large, prospective, and methodologically sound studies that explicitly incorporate inflammatory biomarkers like IL-6 into their primary objectives to validate their clinical relevance in patients undergoing CEA and improve current risk stratification models.

## 5. Conclusions

Crucially, this systematic review exposes a gap in the evidence base: there are currently few available studies explicitly designed to evaluate IL-6 as a clinically actionable biomarker in CEA. Future research must prioritize large, prospective studies and registries that rigorously integrate key inflammatory markers for risk stratification into their primary objectives. It is reasonable to hypothesize that validating the prognostic potential of the IL-6 pathway could refine patient selection and inform personalized anti-inflammatory strategies in the era of anti-IL-6 therapies, and ultimately, provide a more precise prediction and intervention for short- and long-term outcomes on patients undergoing carotid revascularization. In doing so, the field can progress from traditional anatomical and clinical risk models toward a biologically informed precision-medicine framework for managing carotid atherosclerotic disease.

## Data Availability

The original contributions presented in this study are included in the article/Supplementary Materials. Further inquiries can be directed to the corresponding author.

## Supplementary Materials

The following supporting information can be downloaded at: https://www.mdpi.com/article/10.3390/medsci13040325/s1, Table S1: Search query-keywords; Table S2: Study characteristics; Table S3: Population demographics and risk factors; Table S4: Pre-operative characteristics; Table S5: Post-operative outcomes in the first month; Table S6: Long-term outcomes; Table S7: Covariates used in the adjusted models. References [14,15,16,17,18,19,20,21,22,23,24,25,26] are cited in the Supplementary Materials.
