# Supplementary material for: IL-6 and Surgical Outcomes in Carotid Endarterectomy: A Systematic Review
Source: Med Sci (Basel). 2025 Dec 18;13(4):325. doi: 10.3390/medsci13040325 (PMC12734856; doi:10.3390/medsci13040325)
Supplement: Supplementary file 1 [file medsci-13-00325-s001.zip › SupplementalTables_IL6_V15 - edited.pdf]

Supplementary Table S1 – Search query – key words

**Bibliographic source**	**Search term**	**No of reports**<br>1232 total
Pubmed	("interleukin-6" OR "IL-6" OR "IL6" OR "interleukin 6" OR "IL 6")<br>AND<br>(“Endarterectomy” Or “Endarterectomy, Carotid” OR “Carotid Endarterectomy” OR “Carotid Endarterectomies” OR CEA)<br>("Interleukin-6"[Mesh] OR “IL-6” [tiab] OR “interleukin 6” [tiab])<br>AND<br>(“Endarterectomy, Carotid” [MeSH] OR “carotid endarterectomy” [tiab] OR CEA [tiab])	340
ISI	("interleukin-6" OR "IL-6" OR "IL6" OR "interleukin 6" OR "IL 6")<br>AND<br>(“Endarterectomy” Or “Endarterectomy, Carotid” OR “Carotid Endarterectomy” OR “Carotid Endarterectomies” OR CEA)<br>("Interleukin-6"[Mesh] OR “IL-6” [tiab] OR “interleukin 6” [tiab])<br>AND<br>(“Endarterectomy, Carotid” [MeSH] OR “carotid endarterectomy” [tiab] OR CEA [tiab])	350
SCOPUS	("interleukin-6" OR "IL-6" OR "IL6" OR "interleukin 6" OR "IL 6")<br>AND<br>(“Endarterectomy” Or “Endarterectomy, Carotid” OR “Carotid Endarterectomy” OR “Carotid Endarterectomies” OR CEA)<br>("Interleukin-6"[Mesh] OR “IL-6” [tiab] OR “interleukin 6” [tiab])<br>AND	542

	("Endarterectomy, Carotid" [MeSH] OR "carotid endarterectomy" [tiab] OR CEA [tiab])	

Supplemental Table S7. Covariates used in the adjusted models

Author	Covariables used in ajusted models
Arfvidsson et al. [21]	NA
Bountouris et al. [17]	NA
Debing et al. [24]	NA
Grufman et al. [20]	NA
Koutouzis et al. 2009[18]	NA
Palombo et al. [15]	NA
[19]	Model 1 - Diabetes mellitus type 2, C-Reactive protein, Adiponectin, BMI; Model 2 - Gender.
Poredos et al. [23]	NA
Profumo et al. [14]	NA
[16]	NA
[22].	NA
Wang et al. [25]	NA
[26]	Model 1 - Age, gender, BMI, smoking history, alcohol use, hypertension, diabetes, prior stroke, albumin, glucose, triglycerides, total cholesterol, HDL, LDL, Apo A Ⅰ, Apo B, CRP, hemoglobin, procalcitonin, D-dimer, TSH, FT3, FT4, TSHI, TT4RI, TFQI; Model 2 - Smoking history, alcohol use, HDL, TSHI; Model 3 - TSH, TSHI, TT4RI.

BMI – body mass index; CRP – C-reactive protein; FT3 - free triiodothyronine; FT4 - free thyroxine; HDL – high-density lipoproteins; LDL – low-density lipoproteins; TNF-α – tumor necrosis factor alpha; TSH – thyroid-stimulating hormone; TSHI – thyroid-stimulating hormone index; TT4RI - thyrotroph T4 resistance index; TFQI -thyroid feedback quantile-based index.
