# Supplementary material for: IL-6 and Surgical Outcomes in Carotid Endarterectomy: A Systematic Review
Source: Med Sci (Basel). 2025 Dec 18;13(4):325. doi: 10.3390/medsci13040325 (PMC12734856; doi:10.3390/medsci13040325)
Supplement: Supplementary file 1 [file medsci-13-00325-s001.zip › Tables_IL-6_V15 - edited.pdf]

Author	Journal	Publication Year	Study Design	Study Center	Recruitment Time	Sample Size (patients)	No. CEA	GRADE Evaluation
Unic-Stojanovic et al. [22].	Angiology	2016	Prospective cohort	Dedinje Cardiovascular Institute, Serbia	NA	77	77	⊕⊕◯◯ Low
Stein et al. [16]	Journal of Vascular Surgery	2008	Prospective cohort	NA	NA	34	34	⊕◯◯◯ Very Low
Grufman et al.[20]	Journal of Internal Medicine	2014	Prospective cohort	Malmö University Hospital, Sweden	NA	160	160	⊕⊕◯◯ Low
Wang et al. [25]	National Medical Journal of China	2025	Retrospective case-control	Department of Vascular Surgery, Nanjing Drum Tower Hospital, China	January 2019 – October 2023	149	149	⊕⊕◯◯ Low
Profumo et al. [14]	Atherosclerosis	2007	Prospective cohort	University of Rome, "La Sapienza", Italy	NA	43	43	⊕◯◯◯ Very Low
Persson et al. [19]	Atherosclerosis	2012	Prospective cohort	Biobank of Karolinska carotid Endarterectomies (BiKE), Sweden	2002 - 2007	292	292	⊕⊕◯◯ Low
Palombo et al. [15]	European Journal of Vascular and Endovascular Surgery	2007	Prospective randomised study	Unit of Vascular and Endovascular Surgery, Azienda Ospedaliera Univeristaria San Martino, Genova, Italy	February 2006 – September 2006	96	96	⊕⊕◯◯ Low

Zhigao te al. [26]	Frontiers in Cardiovascular Medicine	2024	Retrospective cohort	Nanjing Drum Tower Hospital Clinical College of Nanjing Medical Univeristy, China	May 2019 – May 2023	131	131	⊕⊕◯◯ Low
Bountouris et al. [17]	Angiology	2009	Prospective cohort	Department of Molecular and Clinical Medicine, Univeristy of Gothenburg, Sweden	NA	74	74	⊕⊕◯◯ Low
Debing et al. [24]	Vascular and Endovascular Surgery	2008	Retrospective case-control	Department of Vascular Surgery, University Hospital Brussels, Belgium	June 2004 – September 2006	180	180	⊕⊕◯◯ Low
Arfvidsson et al. [21]	Clinical Chemistry and Laboratory Medicine	2015	Prospective cohort	Umeå University, Sweden	NA	20	20	⊕⊕◯◯ Low
Poredos et al. [23]	Journal of Atherosclerosis and Thrombosis	2017	Prospective cohort	University Medical Centre Ljubljana, Slovenia	NA	21	21	⊕◯◯◯ Very Low
Koutouzis et al. [18]	Acta Neurologica Scandinavica	2009	Prospective cross-sectional	Attikon University Hospital, Greece	NA	119	119	⊕⊕◯◯ Low

Table S2 -Study characteristics

CEA – carotid endartectomy; RA – regional anesthesia; NA – not available

Table S3 - Populations demographics and risk factors

Author	Mean age	Male n(%)	Arterial Hypertension n(%)	Dislipidemia n(%)	Diabetes Mellitus n(%)	Smoking History n(%)	Coronary Artery Disease n(%)	Carotid territory symptoms n(%)
Unic-Stojanovic et al. [22]	65.7	51 (66.2)	74 (96.1)	73 (94.8)	22 (28.6)	47 (61.0)	16 (20.8)	43 (55.8)
Stein et al. [16]	69.0	25 (73.5)	21 (61.8)	16 (47.1)	9 (26.5)	7 (20.6)	10 (29.4)	34 (100.0)
Grufman et al. [20]	69.2	105 (65.6)	121 (75.6)	NA	64 (40.0)	52 (32.5)	NA	87 (54.4)
Wang et al. [25]	68.2	124 (83.2)	111 (74.5)	NA	43 (28.9)	NA	28 (18.8)	NA

Profumo et al. [14]	NA	27 (62.8)	29 (67.4)	12 (27.9)	15 (34.9)	30 (69.8)	NA	NA
Persson et al. [19]	69.7	209 (71.6)	231(79.1)	NA	62 (21.2)	NA	91 (31.2)	218 (74.7)
Palombo et al.[15]	71.5	64 (66.7)	81 (84.4)	50 (52.1)	17 (17.7)	79 (82.3)	NA	33 (34.4)
Zhigao et al. [26]	67.6	107 (81.7)	98(74.8)	NA	44 (33.6)	52 (39.7)	0 (0.0)	68 (51.9)
Bountouris et al. [17]	66.9	55 (74.3)	64 (86.5)	51 (68.9)	25 (33.8)	48 (64.9)	NA	38 (51.4)
Debing et al. [24]	NA	124 (68.9)	146 (81.1)	124 (68.9)	63 (35.0)	70 (38.9)	90 (50.0)	101 (56.1)

Arfvidsson et al. [21]	NA	15 (75.0)	16 (80.0)	NA	1 (5.0)	14 (70.0)	NA	19 (95.0)
Poredos et al. [23]	70.4	12 (57.1)	20 (95.2)	19 (90.5)	8 (38.1)	5 (23.8)	NA	8 (38.1)
Koutouzis et al. [18]	67.0	91 (76.5)	105 (88.2)	NA	33 (27.7)	76 (63.9)	25 (21.0)	62 (52.1)

Legend: NA – unavailable data

Table S4 - Pre-operative characteristics

Author	Contralateral Stenosis n(%)	Antiplatelet Therapy n(%)	Dual Antiplatet Therapy n(%)	Anticoagulation n(%)	General anestesia n(%)	Regional anestesia n(%)
Unic-Stojanovic et al. [22]	7 (9.1)	NA	NA	NA	77 (100.0)	0 (0.0)
Stein et al. [16]	NA	30 (88.2)	NA	NA	NA	NA
Grufman et al.[20]	NA	NA	NA	NA	NA	NA
Wang te al. [25]	NA	NA	NA	NA	NA	NA
Profumo et al. [14]	31 (72.1)	43 (100.0)	NA	NA	NA	NA
Persson et al. [19]	69 (23.6)	278 (95.2)	NA	NA	NA	NA
Palombo et al.[15]	NA	NA	NA	NA	NA	NA
Zhigao et al. [26]	NA	NA	NA	NA	NA	NA
Bountouris et al. [17]	NA	50 (67.6)	NA	NA	NA	NA

Debing et al. [24]	NA	NA	NA	NA	NA	NA
Arfvidsson et al. [21]	9 (45.0)	19 (95.0)	NA	NA	20 (100.0)	0 (0.0)
Poredos et al. [23]	NA	NA	NA	NA	NA	NA
Koutouzis et al. [18]	NA	85 (71.4)	NA	NA	NA	NA

Legend: NA – unavailable data

Table S5 – Post-operative outcomes in the first month

Author	Stroke 30 days n(%)	Stroke/Death 30 days n(%)	Death 30 days n(%)	MI 30 days n(%)	MACCE 30 days n(%)	MACCE definition	Post-operative adverse events 30 days n(%)	Restenosis n(%)
Unic-Stojanovic et al. [22]	1 (1.3)	2 (2.6)	1 (1.3)	NA	3 (3.9)	Stroke and transient ischemic attack	3 (3.9)	2 (2.6)
Stein et al [16]	0 (0.0)	0 (0.0)	0 (0.0)	NA	NA	NA	0 (0.0)	NA
Grufman et al. [20]	NA	NA	NA	NA	NA	NA	NA	0 (0.0)
Wang et al. [25]	NA	NA	NA	NA	NA	NA	NA	NA

Profumo et al. [14]	NA	NA	NA	NA	NA	NA	NA	NA
Persson et al. [19]	NA	NA	NA	NA	NA	NA	NA	NA
Palombo et al.[15]	0 (0.0)	0 (0.0)	0 (0.0)	NA	NA	NA	2 (2.1)	NA
Zhigao et al.[26]	NA	NA	NA	NA	NA	NA	NA	NA
Bountouris et al. [17]	NA	NA	NA	NA	NA	NA	NA	2(2.7)
Debing et al. [24]	NA	NA	NA	NA	NA	NA	NA	0(0.0)

Arfvidsson et al. [21]	NA	NA	NA	NA	NA	NA	NA	NA
Poredos et al. [23]	NA	NA	NA	NA	NA	NA	NA	NA
Koutouzis et al. [18]	2 (1.7)	2 (1.7)	0 (0.0)	2 (1.7)	2 (1.7)	Stroke and myocardial infarction	12 (10.1)	NA

Legend: MACCE – Major Adverse Cardiovascular and Cerebrovascular Events; NA – unavailable data

Table S6 – Long-term outcomes

Author	Other outcomes	Long-term outcomes	Long-term follow-up time	Long-term MI n(%)	Long-term stroke n(%)	Long-term MACE n(%)	Long-term all-cause mortality n(%)
Unic-Stojanovic et al. [22]	3 (3.9) Post-operative cognitive dysfunction	NA	NA	NA	NA	NA	1 (1.3)
Stein et al. [16]	0 (0.0) Post-operative	0 (0.0) Major bleeding,	NA	NA	NA	NA	0 (0.0)

	cognitive dysfunction and surgical hematoma	death or cerebral events					
Grufman et al. [20]	NA	NA	NA	NA	NA	NA	NA
Wang et al. [25]	NA	NA	NA	NA	NA	NA	NA
Profumo et al. [14]	NA	NA	NA	NA	NA	NA	NA
Persson et al. [19]	NA	52 (17.8) (27 from	5.2 years	28 (9.6)	52 (17.8)	73 (25.0)	52 (17.8)

		cardiovascular causes and 25 from other causes)					
Palombo et al. [15]	NA	NA	NA	NA	NA	NA	NA
Zhigao et al. [26]	NA	NA	NA	NA	NA	NA	NA
Boutouris et al. [17]	NA	3 (4.1) (1 underwent a percutaneous coronary intervention and 2	2 years	4 (5.4)	3 (4.1)	15 (20.3)	5 (6.8)

		underwent cere- bral revascularizati on with stenting due to restenosis in the operated carotid artery)					
Debing et al. [24]	NA	NA	NA	NA	NA	NA	NA
Arfvidsson et al. [21]	NA	NA	NA	NA	NA	NA	NA

Poredos et al. [23]	NA	NA	NA	NA	NA	NA	NA
Koutouzis et al. [18]	7 (5.9) Surgical hematoma	NA	44 ± 18 days	NA	NA	NA	NA

Legend: MI – Myocardial infarction; NA – unavailable data
